# STAMP: Extensions to the STADEN sequence analysis package for high throughput interactive microsatellite marker design

**DOI:** 10.1186/1471-2105-10-41

**Published:** 2009-01-30

**Authors:** Lars Kraemer, Bánk Beszteri, Steffi Gäbler-Schwarz, Christoph Held, Florian Leese, Christoph Mayer, Kevin Pöhlmann, Stephan Frickenhaus

**Affiliations:** 1 Institut für Klinische Molekularbiologie, Universität Kiel, Arnold-Heller-Str 3, 24105 Kiel, Germany; 2 Alfred Wegener Institute for Polar and Marine Research, Am Handelshafen 12, 27570 Bremerhaven, Germany; 3 Animal Ecology, Evolution and Biodiversity, Ruhr University, 44780 Bochum, Germany

## Abstract

**Background:**

Microsatellites (MSs) are DNA markers with high analytical power, which are widely used in population genetics, genetic mapping, and forensic studies. Currently available software solutions for high-throughput MS design (i) have shortcomings in detecting and distinguishing imperfect and perfect MSs, (ii) lack often necessary interactive design steps, and (iii) do not allow for the development of primers for multiplex amplifications. We present a set of new tools implemented as extensions to the STADEN package, which provides the backbone functionality for flexible sequence analysis workflows. The possibility to assemble overlapping reads into unique contigs (provided by the base functionality of the STADEN package) is important to avoid developing redundant markers, a feature missing from most other similar tools.

**Results:**

Our extensions to the STADEN package provide the following functionality to facilitate microsatellite (and also minisatellite) marker design: The new modules (i) integrate the state-of-the-art tandem repeat detection and analysis software PHOBOS into workflows, (ii) provide two separate repeat detection steps – with different search criteria – one for masking repetitive regions during assembly of sequencing reads and the other for designing repeat-flanking primers for MS candidate loci, (iii) incorporate the widely used primer design program PRIMER3 into STADEN workflows, enabling the interactive design and visualization of flanking primers for microsatellites, and (iv) provide the functionality to find optimal locus- and primer pair combinations for multiplex primer design. Furthermore, our extensions include a module for storing analysis results in an SQLite database, providing a transparent solution for data access from within as well as from outside of the STADEN Package.

**Conclusion:**

The STADEN package is enhanced by our modules into a highly flexible, high-throughput, interactive tool for conventional and multiplex microsatellite marker design. It gives the user detailed control over the workflow, enabling flexible combinations of manual and automated analysis steps. The software is available under the OpenBSD License [1,2]. The high efficiency of our automated marker design workflow has been confirmed in three microsatellite development projects.

## Background

Microsatellites (MSs), also called simple sequence repeats (SSRs) or short tandem repeats (STRs), are repetitive genomic sequence stretches with repeat unit lengths of 1–6 nucleotides.

The high variability in the number of repeat units of many microsatellites in combination with the high reproducibility of results often make them the marker of choice when fine temporal and/or spatial resolution is needed. Their high analytical power (multilocus, multiallelic, co-dominant, nuclear, single-copy markers) made microsatellites one of the most widely used molecular marker systems in the last two decades [3]. Genotyping of MS loci is usually performed by amplification of the repetitive sequence with primers positioned in more conserved flanking regions. However, the actual design of suitable flanking primers in unknown genomes still accounts for most of the handling time and resource requirements in MS marker design workflows, in particular when multiplex amplification of several loci simultaneously is aimed for.

In order to be most widely applicable, microsatellite marker design workflows should be flexible enough to accept data originating from different sources, such as whole genome sequences, sequences from repeat-enriched genomic libraries, or even EST (expressed sequence tag) data. The input data format can either be text files (e.g, from sequence databases) or trace files obtained directly from the sequencer. A software tool for fast microsatellite marker design should ideally combine high throughput with high flexibility, enabling the user to work with the whole spectrum of possible data sources and to combine different analysis tools in user-definable workflows. While the software should provide the possibility to manually interact with the analysis process at any point, it should be able to perform a number of basic tasks automatically, e.g. base calling, vector and quality clipping, and assembly in case of *de novo *sequencing as well as repeat detection and flanking primer design. The workflow should be applicable to data sets of any size, from individual sequence reads to large genomic data sets. Of great importance is the possibility to manually check and backtrack results of each analysis step, and to introduce changes at any point if required, e.g. shifting primer candidates by a few nucleotides based on sequencing quality information. Last but not least, it should provide the functionality to aid the researcher in finding locus or primer combinations for multiplexing. Although a couple of stand-alone software tools have been developed for automatic MS marker design (MSATCOMMANDER, READ2MARKER, the TROLL pipeline, SSR PRIMER, MSATFINDER, SAT: [4-9]), we found none that could satisfy all the above criteria. Each of these tools has one or more of the following weaknesses: lack of redundancy checks, weak or inconsistent solutions for the repeat detection problem and lack of integration and interactivity. Although, e.g., SAT offers many of the demanded functionalities and the SAT web interface makes it an attractive option, it requires more software dependencies to be fulfilled as a stand-alone tool. These include a database-service, a web-service for the interactive features, a UNIX/LINUX-based compute system capable to run the SAT-scripts, as well as a number of third-party sequence analysis tools (like CAP3, SPUTNIK etc.). The desired integrated features to trace back primer-pairs to sequence-trace-files was found implemented best in the TROLL package. Disadvantages of the latter package concerning repeat detection and lack of integration of primer design, as discussed below, motivated the present work.

Only two of the above mentioned pipelines, the TROLL module for the STADEN package and the SAT pipeline, explicitly address the problem of marker redundancy [6,9]. In most cases, microsatellite markers are developed based on sequencing genomic shotgun libraries, which generally contain redundant sequence fragments. This increases the risk of developing redundant molecular markers if sequenced clones are uncritically treated as representatives of different genomic loci (e.g. loci Ca5 AF277577 & Ca10 AF277582 in [10]). The TROLL module of Martins et al. [6] addresses this issue by using the assembly functionality provided by the STADEN package to detect and assemble overlapping reads as unique contigs. This implementation, however, suffers from the problem that the information of just a single repeat detection step in the workflow is used for both, assembly of sequence reads and primer design for MS candidate loci (see below). SAT [9] uses a similar approach but with an additional SSR detection step after assembling overlapping reads, an important improvement as SSR detection for the purpose of masking for assembly and for primer design require different criteria.

Another weakness of currently available MS marker design pipelines is the repeat detection. While detecting perfect repeats is a relatively straightforward task, which is implemented satisfactorily in several tools, detecting imperfect repeats is a more complex problem and several repeat detection tools perform poorly in this regard. However, detecting imperfect repeats is crucial in microsatellite design workflows for several reasons. First, imperfect repeats can seriously impede the assembly of unique and unrelated contigs which can cluster together if they consist of long and highly similar tandem repeats with only short non-homologous flanking regions. This artifact, which typically arises in repeat-enriched genomic libraries, is avoided by masking repetitive regions during assembly. Second, imperfect MS markers are more common than perfect ones and often represent the major proportion of the markers reported in Molecular Ecology Notes (Molecular Ecology Resources). Since imperfect MSs may suffer from a higher degree of detectable homoplasy than perfect repeats [11-13], it is more difficult to find a simple and appropriate mutation model for them. If, however, a high degree of polymorphism is more important than assumptions on the underlying evolutionary model, imperfect microsatellites are well suited and should thus be correctly recognized in a modern MS design workflow. Of the existing MS marker design pipelines cited above, only SSR PRIMER (using the repeat detection tool SPUTNIK), MSATFINDER (using SPUTNIK or an algorithm designed by the authors) and SAT (also using SPUTNIK) can detect imperfect repeats, however only with a limited accuracy. Finally, even if primers should exclusively be designed for perfect MSs, their reliable automatic detection requires that perfect and imperfect MSs are successfully identified and distinguished by the software, avoiding the risk of choosing perfect subsatellites of larger imperfect satellites as markers.

The novel repeat detection program PHOBOS[14] has been chosen for the STADEN pipeline since it implements a fast, efficient, and highly accurate algorithm to scan molecular sequences for perfect and imperfect tandem repeats without the need to specify a repeat pattern library. Its high accuracy stems from an exact, i.e. non-probabilistic, search algorithm which detects all tandem repeats that match user-defined repeat characteristics. Furthermore, PHOBOS can search for tandem repeats with a unit size of more than 5000 bp, which in the STAMP modules implies that primers can also be designed for minisatellites and tandem repeats with even longer units. Search settings and the output format of PHOBOS can be adjusted in a flexible manner, making it an ideal multipurpose tandem repeat search tool (Mayer in prep.).

Another major incentive motivating our work was the lack of flexibility and interactivity in most MS marker design tools, which simply implement a rigid and for the most part strictly linear workflow. However, an interactive workflow control can improve the usability at several steps during the design process. The TROLL module for the STADEN package [6] demonstrated the power of an approach that uses a modular sequence analysis software as a backbone for a special purpose application, realized by adding some specialized extensions to the basic workflow. Their module integrated the repeat detection tool TROLL  into the PREGAP module of the STADEN package, making it possible to define workflows that combine base calling, vector and quality clipping as well as assembly of sequencing reads with repeat detection. Finally, the results from the pipeline were used in a stand-alone primer design program wrapping PRIMER3 for designing flanking primers.

Besides ignoring imperfect repeats, the integration of TROLL into STADEN is further limited in its usefulness because repeat detection can only be carried out once as part of the pre-processing pipeline for sequence assembly, i.e., no post-assembly repeat detection can be performed. However, the purposes of identifying repeats for assembly and designing flanking primers differ fundamentally. Whereas for masking tandem repeats, a high imperfection should be allowed and all tandem repeats should be searched for, the search criteria for primer design are usually more restrictive with a focus on more perfect repeats with user-defined motifs and length-characteristics.

Furthermore, in the TROLL module, the primer design process was implemented as a stand-alone wrapper script for PRIMER3, an unsatisfying solution concerning integration. Thus, the user has no possibility to interactively validate the primers in the context of their contigs or sequencing reads. This step would be necessary to manually check, e.g., sequence coverage and quality in the primer binding region, or to perform multiple rounds of primer design with the aim of incrementally adding primer candidates from these rounds to a list of most promising primer candidates.

**Figure 1:**
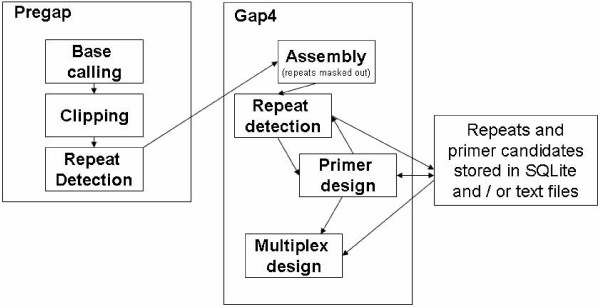
**The diagram illustrates possible workflows for microsatellite marker design in the STADEN package using the STAMP extension modules**.

In view of the major drawbacks of existing stand-alone MS marker design tools we decided to develop a set of extension modules for the STADEN package [15] for a flexible, convenient, and efficient development of MS markers. The STADEN package provides the functionality for data input from multiple sources, base calling, vector and quality clipping, assembly with redundancy checking, interactive contig editor, possibilities to manually and programmatically tag selected regions. Our modules extend STADEN's functionality by the following features: (i) integration of the microsatellite search tool PHOBOS, (ii) independent detection of microsatellites for assembly and marker design with different search parameters, (iii) more flexible integration of repeat detection and primer design steps which can now be performed in an iterative process allowing the user to repeat design steps without having to go through the complete workflow again, (iv) design of MS markers optimized for multiplex PCR. These features are partly visualized in the newly implemented workflows of STAMP depicted in Fig. 1.

In order to improve upon an important weakness of the STADEN package for such applications, namely the difficult programmatic handling of in-memory GAP4 databases, we also implemented an extension providing the possibility to store sequence features (such as tandem repeats and primer candidates) in a more transparent manner in SQLite [16] databases, which allows for an easy exchange and archiving of data in a more common and more flexible file-format compared to the GAP4-data.

## Implementation

All extensions described below were implemented as tcl-modules following the conventions used in the internal modules of the STADEN package.

### PHOBOS module for PREGAP – detection of tandem repeats for assembly

Our first extension is a tcl-module integrating the repeat detection tool PHOBOS into the STADEN pre-processing tool PREGAP. It extends the existing pre-processing pipeline by a tandem repeat detection step. The user has full control over the PHOBOS search parameters and detected tandem repeats can be filtered according to user preferences in the module. Tandem repeats that pass the filter are added as tags to the experiment files for subsequent processing steps.

### Module for GAP4

After the sequence pre-processing step, the experiment files, which include the information on repetitive regions, can be used for the assembly in the GAP4 module of the STADEN package. When using the "Normal shotgun assembly" option, the tagged repeats can be masked out. Either all experiment files or a subset of them can be used as input for the assembly process. After assembly, the individual contigs can be viewed using the contig editor window of GAP4 with tagged regions being marked. If the project started from trace files, the individual traces can even be displayed together with the contigs.

### PHOBOS module for GAP4 – detection of tandem repeats for primer design

**Figure 2:**
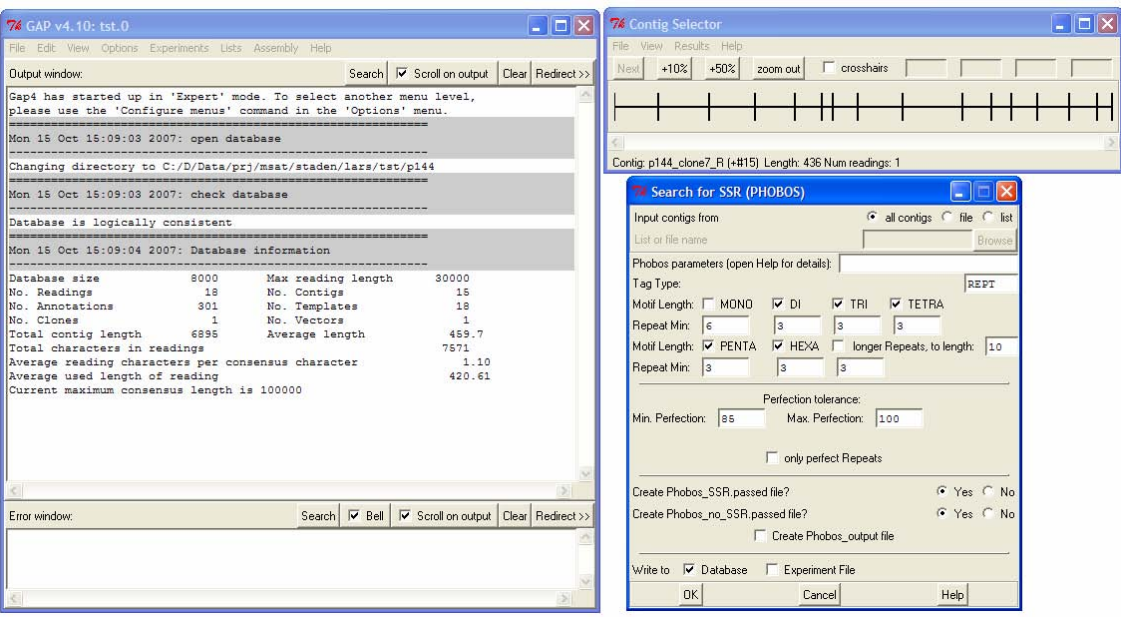
**GAP4 dialog to configure microsatellite detection with PHOBOS**.

On the basis of assembled contigs, a tandem repeat search can be invoked with search parameters independent of a previous search (see Fig. 2). Information on selected tandem repeats can be saved in different forms that can be accessed within STADEN in later analysis steps.

### Module for GAP4 – designing flanking primers

**Figure 3:**
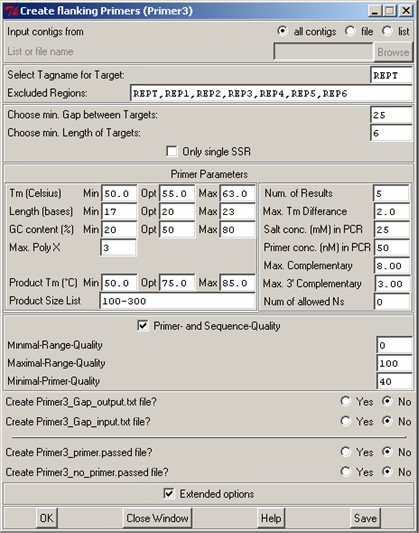
**Dialog to start the design of flanking primers for user defined tags present in a GAP4 database**.

This module integrates PRIMER3 into GAP4 and enables the finding of PCR primer pairs flanking any user specified tag in the GAP4 database. Even though our work focuses on designing primers for tagged tandem repeats, the module was developed with a more general applicability in mind in order to make the functionality of designing flanking primers available for all tagged features in the database/experiment files, see Fig. 3. In this module the user is offered a number of options to choose primer target regions and primer design parameters. The user can specify whether the primer pairs should flank just a single tandem repeat or several tandem repeats for a single PCR product.

**Figure 4:**
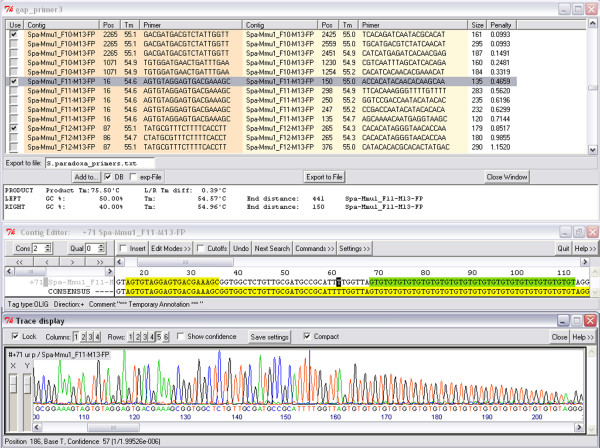
**Combined display of trace, sequence and tags**. From top to bottom: table of results from a flanking primer design process; contig editor window with tagged tandem repeat (green) together with a forward primer candidate (on the left); trace display of the corresponding sequence segment.

After completion of a PRIMER3 run, a table listing the best primer pairs for each contig is being displayed. By clicking on rows of this table, an instance of the contig editor is opened showing the contig corresponding to the primer pair that has been tagged. Check boxes allow the user to manually select or deselect primer pairs for individual contigs (by default, the best primer pair based on PRIMER3 penalties is selected). Fig. 4 gives an example of the combined view of repeat segments, flanking primers, and according trace-files. Subsequently, all checked primer pairs can be written either into the GAP4 database, into experiment files, or an SQLite database. Upon completion of manual checks, the list of selected primer pairs can be exported into a tab delimited text file, useful for e.g., ordering primers.

### Module for GAP4 – selecting primer combinations for multiplex PCR

**Figure 5:**
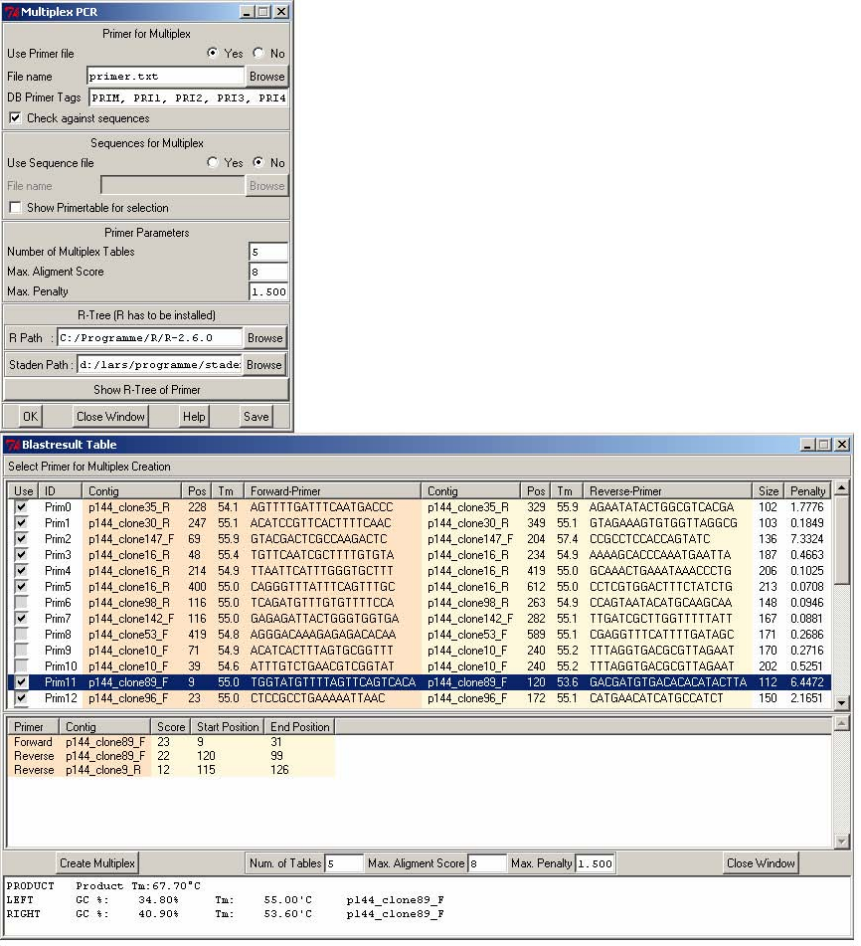
**Top: Multiplex primer design dialog**. Bottom: Table showing similarities between primers and other contigs to detect possible cross-hybridizations.

This module allows the user to search for primer pair combinations which could be used in a multiplex PCR experiment. As input, the module uses a list of candidate primer pairs, which in general come from a previous primer design step. Fig. 5 shows the program dialog for this. To search for compatible primer pair combinations, a score matrix is calculated by aligning all primers with each other. Additionally, BLAST searches of primers against sequences from either the GAP4 database or a user specified FASTA file can be performed, to check for a potential cross-hybridization among different loci and prevent the inclusion of primers that may bind to non-target loci. If the free statistical computing software R [17] is installed, the results of a cluster analysis of primer pairs based on their alignment scores can be visualized. The tree image created using R shows the compatibility between different primer pairs: pairs within near branches are more compatible in a PCR-multiplex, than pairs on more distant branches. The tree is based on a hierarchical cluster analysis of primer-pair alignment scores, calculated on both, the forward and reverse complement strands. The created primer sets can be written as tags into the GAP4 database, experiment files or an SQLite database. It is also possible to export the lists as tab delimited text files.

### SQLite module for GAP4 – storing tagged features

**Figure 6:**
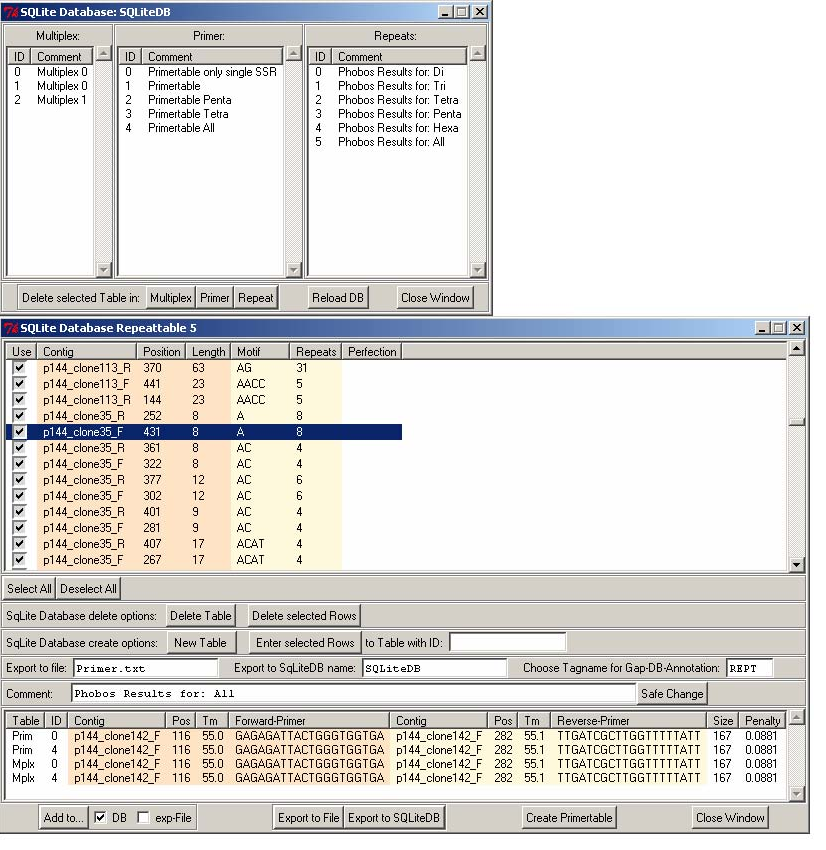
**Overview of SQLite entries and associated flanking primers**. Top: Dialog to load data from SQLite, listing available analysis results. Bottom: Table of tandem repeats and their flanking primers loaded from an SQLite database.

With this module it is possible to store analysis results in SQLite databases. All results created with the other modules listed above (repeat detection, primer design and multiplex design) can (optionally) be written into an SQLite database, as depicted in Fig. 6. This adds a transparent alternative to storing features in experiment files or in-memory databases of the GAP4 module. A data transfer between the in-memory GAP4 database and SQLite databases is possible in both directions. Furthermore, features from the SQLite tables can be added to STADEN experiment files and can be exported as tab-delimited text. A number of further methods to manipulate SQLite databases through a GAP4 interface have been implemented

## Installation

The STAMP extensions can readily be added to an existing installation of the STADEN package by extracting the STAMP archive into the STADEN home folder. Precompiled STAMP archives that contain all necessary binaries (PHOBOS, PRIMER3, NCBI BLAST, SQLite) are available for the Windows, Linux, and MacOS platforms. The archives also include documentation for each of the extension modules in PDF and HTML format as well as tutorials.

## Results and discussion

The pipeline was tested in three microsatellite marker design projects, described in detail in Leese et al. [18], Gäbler et al. (in prep.) and Pöhlmann et al. (in prep.). Table 1 summarizes these results in terms of primer yield.

**Table 1:** Summary and comparison of the results of the four laboratory tests. The "percentage yield" is the ratio of succesful amplifications over the number of loci with tested primers.

	*S. paradoxa*	*P. antarctica*	*N. magellanica*	*N. deaurata*
nr. of reads	167	150	79	87
nr. of contigs	127	119	65	78
nr. of loci with tested primers	22	28	12	12
product size range (bps)	109–332	102–300	117–239	129–293
nr. of succesful amplifications	20	24	10	11

percentage yield	90.91%	85.71%	83.33%	91.67%

### Lab test *Serolis paradoxa*

Microsatellites were enriched using the reporter genome protocol [19,20] with DNA from *Drosophila melanogaster *and *Mus musculus *as enrichment templates [20]. A short-insert genomic library was created, of which inserts from 167 clones were sequenced. Using the GAP4 normal shotgun assembly algorithm and allowing a mismatch percentage of 10% in the flanking regions we found 40 redundant inserts. For the remaining 127 unique inserts we searched for microsatellites with a percentage perfection ≥ 95% using PHOBOS. For 22 appropriate loci, primer pairs were automatically developed using PRIMER3. Twenty of the 22 primer combinations yielded distinct PCR products (91%), which is comparably high. These were analysed for polymorphisms on an ABI3130xl sequencer. Genotyping was performed using the software GENEMAPPER 4.0 (Applied Biosystems). Six of the 20 loci were rejected from analysis due to artifactual allele patterns or unreliable genotyping. Overall success of primer design was therefore as high as 63.6%.

### Lab test *Phaeocystis antarctica*

Total DNA was isolated from *P. antarctica *(Antarctica, 63°15'S, 58°20'W, CSIRO Division of Fisheries, Hobart, Tasmania). Nuclear DNA was purified by ultracentrifugation through a caesium chloride-ethidium bromide density gradient [21] and used to create a microsatellite-enriched library, following a slightly modified protocol based on [21-23]. In total 150 clones were directly sequenced, 127 (85%) contained microsatellite motifs. In eight cases, two inserts overlapped with each other. Thus, altogether 119 non-redundant clones were used for marker design. Repeat detection was performed using a minimum perfection of 85% in PHOBOS. For 28 loci, more than one primer set was automatically designed using PRIMER3. For the majority, only the theoretically best primer set was used for the evaluation. In total, 42 primer sets were tested and PCR products were obtained for 34, yielding usable PCR products in 24 loci (86%).

### Lab test *Nacella magellanica and N. deaurata*

Microsatellite enrichment was conducted as described above (see: Lab test *Serolis paradoxa*) with DNA from *Mus musculus *as enrichment template. For *N. magellanica *14 redundant inserts were found in 79 sequenced clones, allowing a mismatch percentage of 10% in the flanking regions during assemby. The remaining 65 unique inserts yielded 12 suitable loci, for which primers were designed using the multiplex option in PRIMER3 with a Tm of 55°C. For *N. deaurata *9 redundant inserts were found in 87 sequenced clones. The remaining 78 unique inserts resulted in 12 suitable loci. Also here the multiplex option was applied with a Tm of 55°C. For *N. magellanica*, 10 of the 12 loci produced distinct PCR products (83.3%), for *N. deaurata *11 out of 12 (91.7%).

## Conclusion

We developed a set of extension modules to the STADEN package to facilitate the process of microsatellite marker development: integration of PHOBOS in GAP4 and PREGAP, integration of PRIMER3 in GAP4, an interactive primer-analysis for multiplex-PCR, and full support of data-storage in SQLite- and GAP4-databases.

Workflows using these extensions can take full advantage of the rich functionality of the basic STADEN package including the possibility to import data from a variety of sources, to visualize data as well as results, and to export results into several formats. The mentioned alternative tools for MS-design may show sufficient performance and convenience, depending on the demands and preferences of the project and/or the user. The major advantages of STAMP over other currently available tools that serve the same purpose are the consequent integration of state-of-the-art repeat detection and flanking primer design tools into a single, flexibly customizable and interactive analysis environment. The software was successfully used in three laboratory tests, demonstrating that microsatellite marker design led to a high proportion of useful primer pairs. Furthermore, the usage of the new modules strongly increased the efficiency of the process of marker design.

## Availability and requirements

The software can be obtained via FTP-download from . Login as user "anonymous" and with a pseudo-password like me@hotmail.com, change directory to "pub", then to "EDV". Download the file: STAMP-vX.zip, where X is the current version number.

**Project homepage: ** and click "software".

**Operating system(s): **Platform independent

**Programming language: **tcl

**Other requirements: **none/STADEN package

**License: **OpenBSD for the STAMP module.

PHOBOS is copyright protected and free only for academic users.

**Any restrictions to use by non-academics: **For PHOBOS a license needs to be obtained from the author CM.

## Authors' contributions

LK developed the extension modules under the coordination of SF and BB. BB contributed prototype scripts in the initial development phase and drafted the manuscript. All authors contributed to writing the manuscript. FL, CH, and CM advised on practical and theoretical aspects of microsatellite marker design. FL, LK, SGS, and KP performed the laboratory tests and provided user feedback during development. All authors read and approved the final manuscript.
